# Home-based portable fNIRS-derived cortical laterality correlates with impairment and function in chronic stroke

**DOI:** 10.3389/fnhum.2022.1023246

**Published:** 2022-12-09

**Authors:** Christopher Lee Friesen, Michael Lawrence, Tony Gerald Joseph Ingram, Shaun Gregory Boe

**Affiliations:** ^1^Laboratory for Brain Recovery and Function, Dalhousie University, Halifax, NS, Canada; ^2^Axem Neurotechnology, Halifax, NS, Canada; ^3^School of Physiotherapy, Dalhousie University, Halifax, NS, Canada; ^4^School of Health and Human Performance, Dalhousie University, Halifax, NS, Canada; ^5^Department of Psychology and Neuroscience, Dalhousie University, Halifax, NS, Canada

**Keywords:** functional near-infrared spectroscopy, motor cortex, neuroimaging, rehabilitation, stroke

## Abstract

**Introduction:**

Improved understanding of the relationship between post-stroke rehabilitation interventions and functional motor outcomes could result in improvements in the efficacy of post-stroke physical rehabilitation. The laterality of motor cortex activity (M1-LAT) during paretic upper-extremity movement has been documented as a useful biomarker of post-stroke motor recovery. However, the expensive, labor intensive, and laboratory-based equipment required to take measurements of M1-LAT limit its potential clinical utility in improving post-stroke physical rehabilitation. The present study tested the ability of a mobile functional near-infrared spectroscopy (fNIRS) system (designed to enable independent measurement by stroke survivors) to measure cerebral hemodynamics at the motor cortex in the homes of chronic stroke survivors.

**Methods:**

Eleven chronic stroke survivors, ranging widely in their level of upper-extremity motor deficit, used their stroke-affected upper-extremity to perform a simple unilateral movement protocol in their homes while a wireless prototype fNIRS headband took measurements at the motor cortex. Measures of participants' upper-extremity impairment and function were taken.

**Results:**

Participants demonstrated either a typically lateralized response, with an increase in contralateral relative oxyhemoglobin (ΔHbO), or response showing a bilateral pattern of increase in ΔHbO during the motor task. During the simple unilateral task, M1-LAT correlated significantly with measures of both upper-extremity impairment and function, indicating that participants with more severe motor deficits had more a more atypical (i.e., bilateral) pattern of lateralization.

**Discussion:**

These results indicate it is feasible to gain M1-LAT measures from stroke survivors in their homes using fNIRS. These findings represent a preliminary step toward the goals of using ergonomic functional neuroimaging to improve post-stroke rehabilitative care, via the capture of neural biomarkers of post-stroke motor recovery, and/or via use as part of an accessible rehabilitation brain-computer-interface.

## Introduction

It has become part of the consensus in clinical research that the investigation of post-stroke motor recovery must include and make use of neural biomarkers of recovery (Boyd et al., 2017; Bernhardt et al., 2019; Cramer, 2020). This call for the use of biomarkers in post-stroke motor recovery is an acknowledgment that our imperfect understanding of its mechanisms hold back our ability to optimize the delivery of post-stroke physical rehabilitation, as well as to develop new rehabilitation interventions. And indeed, our imperfect grasp on the causal mechanisms at play is particularly frustrating and troubling in the face of studies showing, for example, that nearly 50% of patients fail to significantly benefit from upper-extremity inpatient rehabilitation (Houwink et al., 2013); or to help us better understand why only 12% of stroke survivors with upper-extremity deficits fully regain its functional capacity (Kwakkel and Kollen, 2013). Moreover, given the fact that clinical trials with a biological rationale have been shown to outperform those without one (Borschmann et al., 2018), the clamoring of the clinical and research community for more measures from the nervous system during rehabilitation, which might in time enable a better understanding of the impact of our interventions, is completely understandable.

The persisting uncertainty around the mechanisms of post-stroke motor recovery is especially concerning when considered alongside the high cost of rehabilitation to the health care system (in the U.S. alone over $41 billion is spent on post-stroke rehabilitation; Ovbiagele et al., 2013). And indeed, unfortunately the high cost of rehabilitation itself also limits our ability to take advantage of what we do understand about post-stroke recovery—for example, our inability to meet established standards on the amount of rehabilitation volume stroke survivors should receive (Hayward and Brauer, 2015; SSNAP, 2015), in light of pre-clinical literature which suggests that the amount of volume prescribed by these standards is itself insufficient to drive maximal cortical re-organization in the motor system (Nudo and Milliken, 1996). And while the Queen Square programme—which produced uniquely strong positive results with very large volumes of rehabilitation in chronic stroke survivors (Ward et al., 2019)—is an encouraging counterpoint to this trend, it is unlikely that scaling this model alone will bring about the type of transformative change needed to how we approach post-stroke recovery, given the high costs and inconsistent effectiveness of rehabilitation interventions. Given this, it seems that improving our understanding of stroke survivors' likelihood of responding to a given intervention (considering aspects such as stroke type, chronicity, modality, and overall volume), is required to significantly improve the standard of care in post-stroke rehabilitation.

Seen another way, the attempt to increase the volume of rehabilitation patients receive, as demonstrated in the Queen Square programme, as well as the integration of neural biomarkers into stroke recovery research, might both be viewed as an attempt to overcome the proportional recovery rule of post-stroke motor recovery—which controversially posits that survivors of stroke can be expected to gain back ~70% of the difference between their acute post-stroke function (for a given measure) and typical function, regardless of rehabilitation interventions. This model and its implicit implications (i.e., that stroke survivor's recovery potential operates by some “rule” independent of rehabilitation) challenge the utility of deploying more public health system resources on rehabilitation as an “investment,” given rehabilitation's highly variable return on investment over-and-above the progress that can be expected regardless of intervention. Despite challenges to this model's particular formulation [due to statistical coupling (Hawe et al., 2019; Hope et al., 2019) and its dependence on the types of assessment measures used (Senesh and Reinkensmeyer, 2019)], if nothing else it persists as a more general skepticism—a belief that post-stroke physical rehabilitation can only marginally improve stroke survivor's motor recovery. However, several recent studies suggest that by utilizing central nervous system-based biomarkers the proportional recovery model can not only be broken down but overcome. Firstly, there is pre-clinical work (Jeffers et al., 2018b; van der Vliet et al., 2020) showing that an individual animal's rehabilitation needs can be better characterized by using neural biomarkers (in this case information on the nature of an animal's lesion). And recent retrospective analyses in humans suggest these limitations to the proportional recovery rule may generalize across species, finding that sophisticated modeling of stroke survivor's upper-extremity recovery reveals a reality more complex than the proportional recovery rule's simple heuristic (Senesh and Reinkensmeyer, 2019; van der Vliet et al., 2020). Moreover, the authors of one of these studies suggested it may be possible to target patients who are more likely to make outsized gains in motor recovery, over and above that which they may make by being provided with the standard of care, by identifying participants with latent corticospinal tract capacity (Senesh and Reinkensmeyer, 2019). Indeed, the improvement in our ability to model and understand the nuances of post-stroke recovery, together with our ability to better capture information from the nervous system, is surely the future of stroke rehabilitation. One impressive early example of this being the PREP algorithm's ability to increase the efficiency of rehabilitation (in this case leading to decreased length of stay at an inpatient rehabilitation facility with no decrease in functional outcomes; Stinear et al., 2007, 2012).

One candidate biomarker of motor recovery in stroke is the ratio of activity between the primary motor cortices during movement of one's paretic limb (i.e., motor cortex laterality; M1-LAT). Using functional magnetic resonance imaging (fMRI) (Marshall et al., 2000; Tombari et al., 2004; Daly et al., 2014), functional near-infrared spectroscopy (fNIRS) (Takeda et al., 2007; Delorme et al., 2017), or electroencephalography (EEG) (Kaiser et al., 2012), it has been shown that deviation from a typical contralateralized pattern of M1-LAT corresponds to worse upper-extremity movement deficits. Moreover, fNIRS studies have also found that the inverse pattern (i.e., a departure from the typically symmetrical pattern of M1-LAT) corresponds to worse gait abilities (Miyai et al., 2006). Studies have also shown that M1-LAT is not only associated with, but predictive of future function as well as one's response to rehabilitation: it has been found to outperform functional status in predicting motor deficits 3 months (Nhan et al., 2004), 6 months (Rehme et al., 2015), and 1 year (Loubinoux et al., 2007) following incidence of stroke, as well as being predictive of a stroke survivor's response to 1 month of rehabilitation (Quinlan et al., 2015).

The mechanistic story underlying the origin of M1-LAT's relevance in post-stroke recovery is still an active area of investigation. There is empirical support for several mechanistic accounts—which, while not being mutually exclusive, have not to date cohered into a single unified framework. For instance, there are empirically supported accounts that place the emphasis on functional cortical changes. Specifically, that reduced ipsilesional M1 activity is due to maladaptive interhemispheric inhibition, whereby a disinhibited contralesional M1 inhibits ipsilesional M1 (Rehme and Grefkes, 2013; Volz et al., 2015). Conversely another hypothesis holds that altered intrahemispheric inhibition, whereby reduced excitatory input from the ipsilesional supplementary motor area to ipsilesional M1 is responsible for reduced ipsilesional M1 output (Grefkes et al., 2008; Rehme et al., 2011). There are also mechanistic explanations focusing on the status of the descending tracts of the motor system. In particular, findings that show a synergy between the structural and functional status of the ipsilesional corticospinal tract correlate with upper-extremity motor recovery (Stinear et al., 2007; Guder et al., 2021) comport with the finding that typical M1-LAT is associated with better post-stroke motor recovery (Schulz et al., 2021).

Despite the lack of clarity as to which of these mechanistic accounts may be causing or be caused by the others, one might imagine how widespread, longitudinal collection of a predictive biomarker like M1-LAT could aid in optimizing how rehabilitation resources are deployed. A major barrier in realizing this potential is that at present it is expensive and labor-intensive to take any relevant biomarkers of post-stroke motor recovery. All the studies mentioned above that utilized M1-LAT to characterize and/or predict recovery used either fMRI or laboratory-based fNIRS/EEG equipment to take their M1-LAT measurements. This is why leaders of the research community have acknowledged the inability of laboratory-based systems to be deployed at the scale required for widespread collection of biomarkers in rehabilitation as a central challenge to overcome in the use of any potential biomarkers of stroke recovery (Boyd et al., 2017). Thus, there is a need to develop technology that makes the collection of relevant biomarkers easier. While most of the literature on the relationship between M1-LAT and post-stroke motor recovery has been based on fMRI measurements, the high cost, specialized staff, and lengthy set-up time required to take these measurements limits its clinical utility in this domain. Moreover, the fact that wet, head-cap-based EEG systems require a lengthy set-up process by a trained experimenter, as well as the difficulty in gaining spatially specific measurements with dry and/or non-full-headcap based EEG (due to the smearing of electrical activity at the scalp; Müller-Gerking et al., 1999) limit its clinical utility for this purpose as well.

Functional near-infrared spectroscopy is a non-invasive functional neuroimaging modality that leverages the differential absorption coefficients of oxyhemoglobin (HbO) and deoxyhemoglobin (Hb) to infer neural activity via the measurement of relative oxy- and deoxyhemoglobin. Specifically, fNIRS measures changes in HbO (ΔHbO) and Hb (ΔHb) via the emission and detection of multiple wavelengths of light. The light weight and robust nature of the components required for fNIRS (which has been and will likely continue to increase, given the ubiquity of LEDs and photo diodes in many common electronic devices) make it well-suited to neuroergonomic use cases where spatially-specific cortical information is of interest (Ferrari and Quaresima, 2012; Ayaz and Dehais, 2021). Indeed, the use of fNIRS in the field of neuroergonomics has and by all measures will continue to grow in popularity (Naseer and Hong, 2015; Thomas and Nam, 2020). The suitability of fNIRS to such tasks is best considered in reference to all other options (Pinti et al., 2020): compared with fMRI and wet EEG, fNIRS offers a measurement method and form factor that are quicker to deploy; while compared with dry EEG, fNIRS' ability to take spatially-specific cortical measurements (given its use of the hemodynamic response as opposed to the measurement of electrical potentials at the scalp—which sufferings a smearing effect rendering spatially-specific information challenging to obtain in the absence of many low-impedance measurement locations), make it well-suited where spatially-specific cortical signals are to be captured in a scenario requiring an easy to deploy and ergonomic measurement method.

Given that fNIRS has also been shown to be capable of characterizing the cortical signatures of various motor tasks (Leff et al., 2011), of taking measurements of post-stroke motor cortex laterality (Takeda et al., 2007; Delorme et al., 2017), and can be made portable (Pinti et al., 2018), fNIRS may be a viable modality to increase the clinical utility of M1-LAT in post-stroke physical rehabilitation. Specifically, the use of ergonomic fNIRS devices to capture neural biomarkers of post-stroke motor recovery, and/or to be used as a part of more accessible rehabilitation brain-computer-interfaces has the potential to help clinicians better understand and thereby optimize post-stroke rehabilitation. And furthermore, such fNIRS devices could also be used in the development of brain-computer-interface systems designed to enhance post-stroke rehabilitation.

The present study tests the ability of a prototype fNIRS headband to take measurements of M1-LAT (during upper-extremity movements) from chronic stroke survivors in their homes, and moreover examines the relationship between these measures of M1-LAT and measures of upper-extremity impairment and function. Specifically, the study hypothesizes that measures of M1-LAT taken via fNIRS will correlate with measures of upper-extremity impairment and function. The prototype fNIRS headband was designed to measure cerebral hemodynamics from the sensorimotor cortices, and to enable independent placement by a stroke survivor; the headband is synchronized to a tablet-based app which guides stroke survivors through upper-extremity rehabilitation exercises while fNIRS measurements are taken. This work contributes to the ultimate goal of demonstrating that neural biomarkers of post-stroke motor recovery can be taken outside the laboratory, with easy-to-use equipment that might one day enable an improvement of the standard of care for post-stroke physical rehabilitation.

## Materials and methods

### Participants

Twelve chronic stroke survivors (three women; age: *M* = 62.2, *SD* = 11.8; months since most recent stroke: *M* = 63.3, *SD* = 41.5) were recruited from the community (see Supplementary Table 1 for full participant characteristics including stroke side, handedness, and history of past stroke). The study received ethical approval from Veritas IRB. Inclusion criteria required participants to self-report that they had previously experienced at least one stroke and were currently experiencing some level of hemiparesis as a result; it also required them to score ≥16 on the Mini-Mental State Examination (MMSE) (Folstein et al., 1975) or (if they failed to do so) ≥19 on the Cognitive Assessment for Stroke Patients (CASP) (Barnay et al., 2014; Benaim et al., 2015; Park et al., 2017).

### Cognitive testing

Participants were first asked to complete the MMSE. The MMSE contains 30 questions designed to identify whether individuals are experiencing serious cognitive impairment. The cut off for participation used in the present study was the level set for administration of the Stroke Impact Scale (SIS) (Sullivan, 2014) [see Section Hand Domain of the Stroke Impact Scale (SIS-Hand)] to ensure participants are capable of following instructions and providing valid feedback. While the MMSE was utilized due to its clinical ubiquity (particularly in the United States), a low score on the MMSE may be due to aphasia rather than cognitive impairment; thus if a participant scored < 17 on the MMSE, they were asked to complete the CASP, a cognitive assessment designed for stroke survivors with language impairments (Barnay et al., 2014; Benaim et al., 2015). The cut off of 19 chosen for the CASP (as discussed in Section Participants) was meant to harmonize to the MMSE cut off of 16 chosen for the present study (Park et al., 2017; Crivelli et al., 2018).

### Fugl-Meyer short form upper extremity section

Participants had their hemiparetic upper-extremity function characterized using the 12-item upper-extremity Fugl-Meyer (FM-12)—a series of six standardized tasks designed to span a wide range of difficulty levels for stroke survivors with upper extremity hemiparesis—as it has been shown to adequately assess motor function of stroke patients while subjecting patients to minimal assessment time (< 10 min; Hsieh et al., 2007; Chen et al., 2014). Assessment of FM-12 items was administered using previously established standardized procedures (Murphy et al., 2011; Sullivan et al., 2011).

### Hand domain of the Stroke Impact Scale (SIS-Hand)

Participants were asked to complete the SIS-Hand, which comprises five questions pertaining to their perception of their stroke-affected hand function. The SIS is a well-established stroke specific health status measure that is appropriate for self-report and does not require training to administer (Sullivan, 2014).

### Motor task

**Figure 1:**
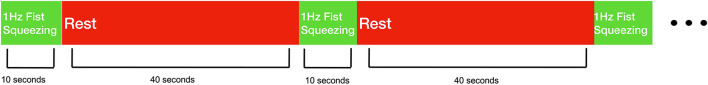
Behavioral task design. Stroke survivors performed 10 trials of 1 Hz fist squeezing. Fist squeezing trials were 10 s, while rest periods were 40 s.

**Figure 2:**
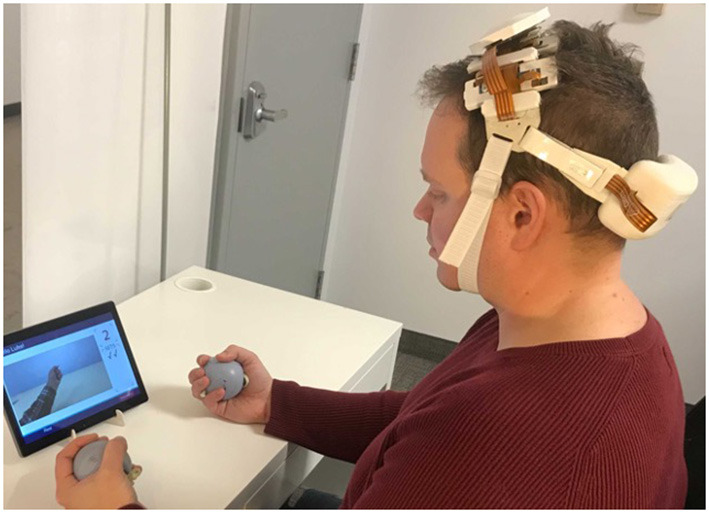
Illustration of the experimental set up for the present study. Participants wore the prototype fNIRS headband while a first-person video of a fist squeezing task was played on a tablet. Participants were asked to follow along with the movements in the video as best they were able.

Experimental sessions took place in the homes of the recruited stroke survivors. All participants performed a fist squeezing task on the side they reported experiencing upper-extremity hemiplegia. Participants were instructed to follow along with a first-person video displayed on a tablet on the table in front of them—the video showed a fist squeezing task being performed with an exercise ball, with the ball being squeezed at ~1 Hz. Participants were also provided an exercise ball, which some chose to use but some were not able to make use of because of their deficit in hand function. Participants performed 10 trials where they were instructed to squeeze along with the video in this manner for 10 s after which they were asked to rest for 40 s (see Figure 1). Participants were asked to perform this task as best they could, and to utilize motor imagery (i.e., the mental rehearsal of movement) if they are unable to complete the movement physically. While the fist squeezing task was being performed, measurements at the lateral motor cortex were taken with a prototype fNIRS headband (see Figure 2).

### Prototype fNIRS headband

**Figure 3:**
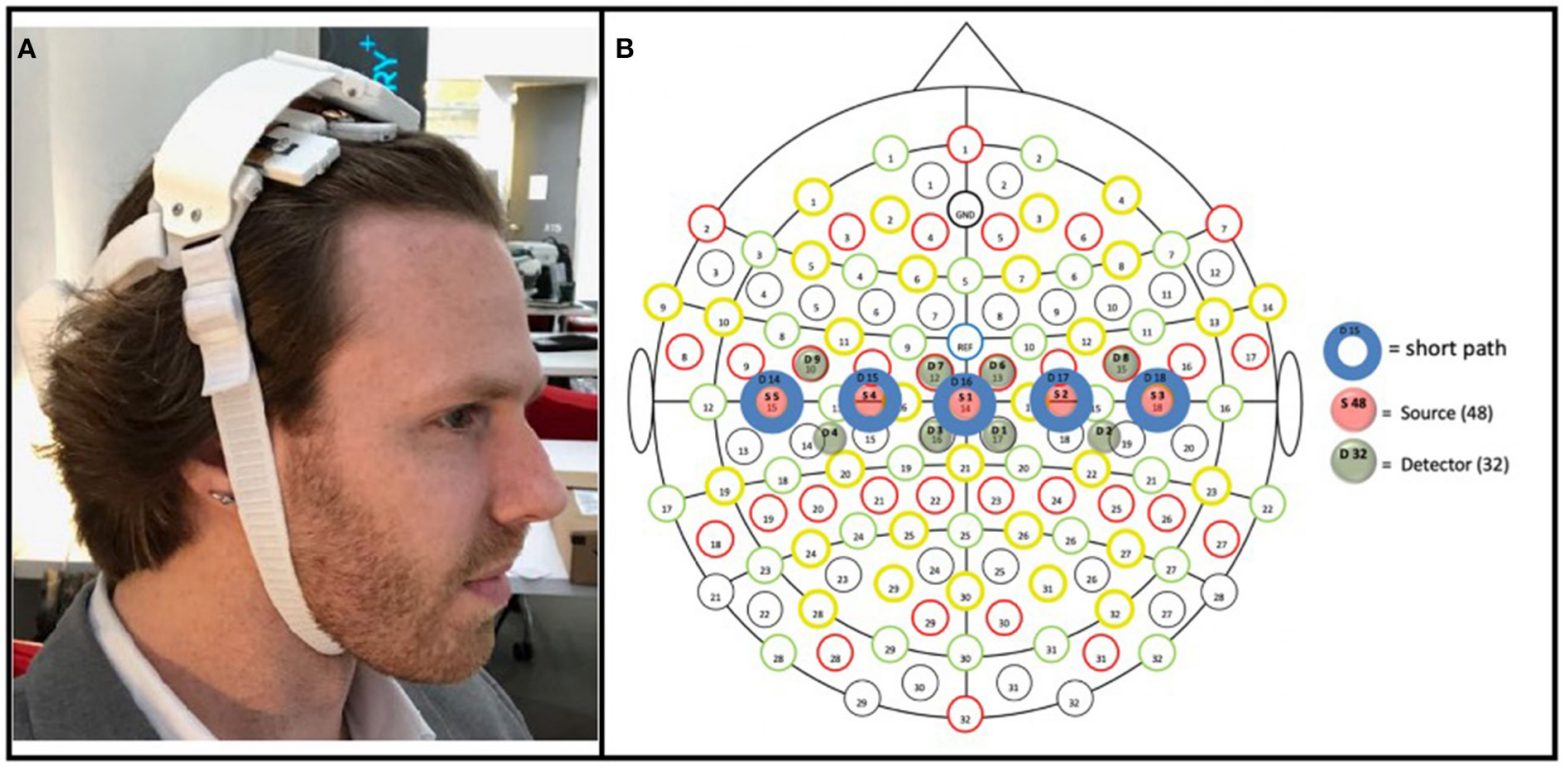
**(A)** Prototype fNIRS headband used in the present study. As pictured, the headband's battery was housed on the back of the head, connected by a strap to the main headband unit; a controller PCB sat above the flexible measurement components (which interfaced with the hair/scalp); and the device was held in place by a chin strap. **(B)** Array of optical components included in the prototype fNIRS headband. Blue circles (D14–D18) represent detectors; green circles (D1–D9) represent long-path LEDs (positioned 3 cm from adjacent detectors); red circles represent short-path (positioned 8 mm from its adjacent detector). The three medial detectors (D15, D16, and D17), being 3 cm from four long-path LEDs (e.g., D15 was 3 cm from D3, D4, D7, and D9), thus enabled four measurement locations each, with the two detectors on either end (D14 and D18), being 3 cm from two long-path LEDs, enabled two measurement locations; resulting in a total of 16 measurement locations.

The prototype fNIRS headband used in the present study was powered by a lithium-ion battery attached to a headband of optical components (see Friesen et al., 2022 in which the device used here was directly compared to an established, research-grade fNIRS system during a motor task); the headband utilized Bluetooth low energy and supports a 8 × 2 grid of 16 unique cerebral hemodynamic measurement locations (see Figure 3B)—thus the fNIRS headband was entirely wireless, with no fiber optic cable, data transmission wires, or the need to clip the power supply to the body (as other fNIRS devices that measure through hair have employed; Piper et al., 2014) increasing the ease of set-up in uncontrolled environments (in this case in the homes of stroke survivors). The headband is meant to be worn at the apex of the head (i.e., approximately where over-the-ear headphones sit) to enable measurement of the brain's sensorimotor region, with the lateral measurement locations overlaying C3 and C4 of the international 10–20 system, which have been shown to overlay the portion of the motor cortex associated with upper-extremity movement (Homan, 1988). The components and measurement locations enabled by the prototype fNIRS headband are illustrated in Figure 3B. The headband contained both long-path (3 cm from the detector; 745 and 850 nm), as well as short-path (8 mm from the detector; 735 and 850 nm) (Sato et al., 2016) channels. The LEDs supporting long-path channels were attached to the headband by individually articulating springs (Figure 3A), allowing the headband to adjust to the shape of users' heads in the sagittal plane, while the use of a flexible central band (which contained the detectors and short-path LEDs) allowed for adjustment in the coronal plane. Importantly, all optical components (i.e., LEDs and silicon photodiodes) were butt coupled to light pipes which enabled light transmission to and from the scalp. These light pipes were of a relatively low durometer (i.e., are softer) compared to traditional fiber optic cable, which allows them to be “worked through hair” by simply shuffling the headband back and forth on the head, whilst remaining comfortable despite making secure contact with the scalp. All these design features (a flexible, one-size-fits all band, which can be manipulated through hair by the person donning the device) in concert allow for a quick and simple device set up—in the present study it allowed the experimenter to set up the device in ~1 min.

While the headband was designed with independent use in mind, in the present study the headband was placed by the experimenter, with its center detector (i.e., item S1 in Figure 3B) positioned at CZ according to the international 10–20 system.

### fNIRS acquisition and pre-processing

The prototype fNIRS headband used in the present study had a system-wide sample rate of 5.4 Hz. Pre-processing procedures were applied as described previously (Friesen et al., 2022; see Supplementary Figure 1 for an overview). Briefly, temporal derivative distribution repair (Fishburn et al., 2019) was applied to all signals. Three sets of inter-channel delays were calculated from 850 nm data which had been band passed to the cardiac pulse (0.5–1.5 Hz), respiration (0.15–0.3 Hz), and Mayer Wave (0.05–0.15 Hz) bands. All data were then transformed to ΔHbO and ΔHb using the modified Beer Lambert equations (Baker et al., 2014). Data from the short-paths was then submitted to a structural equation model (SEM) to estimate latent common influences between the short-path channel in four permutations—once using the unfiltered ΔHbO and ΔHb data, then three more times, using ΔHbO and ΔHb data filtered to the cardiac pulse, respiration, and Mayer Wave bands, respectively; on these new three filtered versions of the ΔHbO and ΔHb data, the previously estimated delays were removed by interpolation prior to estimation of the latent common signal. The resulting four latent common signals were then regressed out of all long-path and short-path ΔHbO and ΔHb channels. Next, information from each long-path channel's associated local short-path was regressed out of the long-path data—this again was conducted both using the unfiltered short-path data, as well as short-path data which had been filtered to the cardiac pulse, respiration, and Mayer Wave bands (which again had had their band-specific delays removed by linear interpolation). And finally, all long-path channels were filtered to the frequency band containing the BOLD response (0.01–0.1 Hz) and Correlation-Based Signal Improvement used to subtract any residual noise from the ΔHbO data, via its method of maximizing the negative correlation between ΔHbO and ΔHb (meaning the ΔHbO data passing through this transform factors in the corresponding ΔHb data, and thus that only ΔHbO data need be considered in subsequent analyses; Cui et al., 2010). Lastly, the resulting ΔHbO data was baseline corrected (to the moment of task onset).

### fNIRS M1-LAT analysis

After pre-processing, for each movement trial, linear slope was fit to the ΔHbO values observed during the 10 s task window at each measurement location, then the difference between homotopic locations (contralesional minus ipsilesional) was computed, resulting in eight unique M1-LAT values (i.e., 16 locations where each M1-LAT value represents the ratio between two of the measurement locations). Insofar as the task timing should (and does, see Supplementary Figure 2) yield relatively monotonically-increasing BOLD time series during the task period, this difference-between-linear-slopes measure serves to quantify the hemispheric asymmetry in task-related activity, and henceforward we refer to this quantity as “M1-LAT” (M1 Lateralization).

To evaluate the relationships between M1-LAT and our measures of post-stroke upper-extremity impairment (FM-12) and function (SIS-Hand), we constructed a SEM. Structural equation models are the more powerful successor to standard regression models, whereby the dependence between outcomes can be evaluated in the presence of both measurement noise and hierarchical structure (Schreiber et al., 2006; Weston and Gore, 2006).

**Figure 4:**
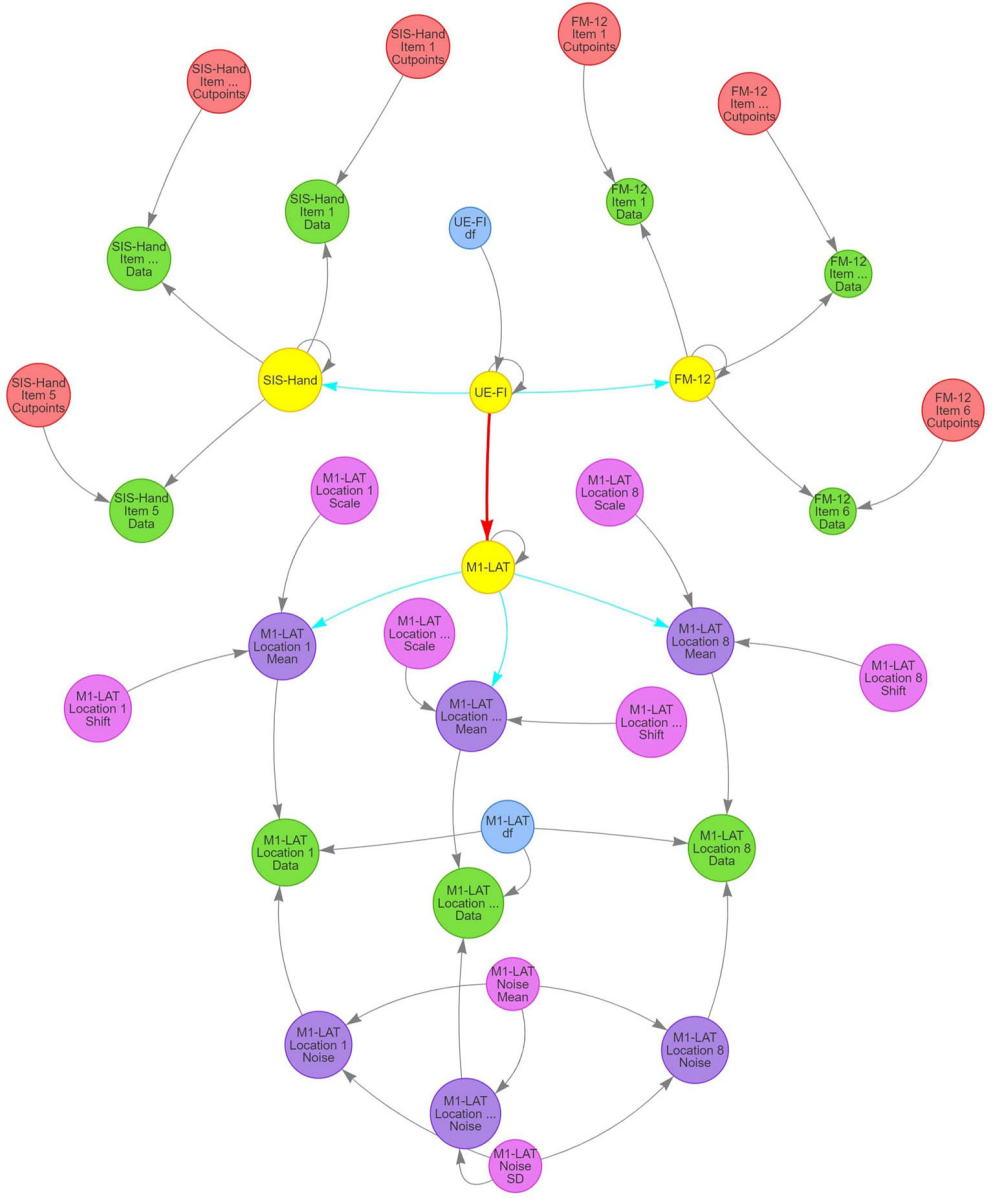
Structure of SEM model used in present study to investigate the relationship between M1-LAT and post-stroke upper-extremity movement. Gray arrows signify deterministic links between constructs, blue arrows signify positively-constrained correlation parameters, and red arrows signify unconstrained correlation parameters. Critically, pink circles represent the main latent variables representing all functional assessments performed in the present study (i.e., FM-12 and SIS), as well as the latent UE-IF variable.

The model's structure (Figure 4) comprises the expectation of a latent (i.e., unobserved, but eventually informed/constrained by the data) trait representing upper-extremity function and impairment (UE-IF in Figure 4). This latent trait employs a zero-mean/unit-variance Student-*t* distribution, with an additional third parameter representing the degrees-of-freedom (to model the heavy-tailedness of the distribution) that varies among participants. Next, the model expressed that the latent UE-IF trait influences three additional latent sub-traits (SIS-Hand, FM-12, and M1-LAT in Figure 4). Each of these sub-traits is modeled with independent normally-distributed residual variance, where the magnitude of influence from the latent to the sub-traits was encoded to enable interpretation as a correlation coefficient between each sub-trait and latent UE-IF (i.e., by a multiplicative quantity whose absolute value ranged from 0 to 1, paired with a magnitude of residual variance ensuring the two combine to yield an expectation of unit variance for each sub-trait). The influence of latent UE-IF was constrained to be positive for both latent SIS-Hand and FM-12 sub-traits (blue arrows in Figure 4) and allowed the full range from −1 to +1 for the M1-LAT sub-trait (red arrow in Figure 4); collectively these constraints ensure that the latent UE-IF trait reflects the information common to the latent FM-12 and SIS-Hand sub-traits, while imposing no a-priori beliefs on the magnitude nor sign of any relationship between latent M1-LAT and the information common to the latent FM-12 and latent SIS-Hand sub-traits.

Latent FM-12 and latent SIS-Hand sub-traits were then connected with their respective item-by-item data for each participant using an ordinal likelihood with a cumulative normal propensity model. Under this approach, the same normal (unit variance and mean determined by that participant's latent sub-trait) was used for the propensity parameter for responses to all items, but items were permitted to vary in the location of the ordinal cut-points and a given item's cut-points were the same for all participants (thereby permitting both differential bias and sensitivity among items). Latent M1-LAT was connected to the M1-LAT data through a set of intermediate latent variables, one for each of the eight fNIRS measurement locations (M1-LAT Location 1–8 Mean in Figure 4; see Supplementary Figure 4.1 for each location's posterior), and the relationship between latent M1-LAT and these intermediary location variables was positively constrained (reflecting the assumption that individual locations all contain varying degrees of common information); these eight latent variables were combined with parameters associated with the shift and scaling necessary to transform the zero-mean/unit-variance distribution of participant's latent quantities to a mean and across-participant variability reflective of the data (M1-LAT Location 1–8 Shift/Scale in Figure 4; see Supplementary Figures 4.2, 4.3 for all priors and posteriors). Moreover, each combination of participant and location were furthermore combined with a parameter encoding the magnitude (standard deviation) of trial-to-trial noise manifest in the data (M1-LAT Location 1–8 Noise in Figure 4), with partial-pooling of values across participants and locations through a hierarchical normal model (implemented on the log-variance scale) with a latent mean and standard deviation (M1-LAT Noise Mean/SD in Figure 4). Finally, using a given combination of participant and location's mean and noise, the trial-by-trial data were modeled by a Student-*t* distributed likelihood with a single DF parameter common to all participants and locations (M1-LAT DF in Figure 4; see Supplementary Figures 4.4, 4.5 for priors and posteriors).

We then sought Bayesian estimation of parameters manifest in this model structure, to derive the relative credibility of various values of each given both the data and whatever prior information we might have about said parameters. While the model has many parameters (e.g., the value of a given latent trait for a given participant can be considered a parameter), the structure described above constrains the vast majority. For all SEM “influence” parameters, uninformed flat priors were used. For all cutpoints, we use the “induced Dirichlet” prior (Betancourt, 2019; Bürkner and Vuorre, 2019). Both Student-*t* degrees-of-freedom parameters received a prior equivalent to a parabola peaked at 15 and ranging from 0 to 30 [achieved as DF/30 ~ beta (2, 2)]. A weakly-informed/data-driven prior was achieved for the remaining fNIRS-related parameters by pre-scaling the data (subtracting a robust estimator of the mean then dividing by a robust estimator of the standard deviation) then using zero-mean/unit-variance normal priors for all mean-encoding parameters and a zero-avoiding/unit-scale (shape = 2, scale = 1) Weibull prior for all standard-deviation-encoding parameters.

The model and priors were expressed in Stan (Gelman et al., 2015), permitting use of the cmdstan Markov Chain Monte Carlo sampler to generate posterior samples reflecting the posterior probability distributions on the model parameters given the model structure, priors, and observed data. Diagnostics for all sampling runs were evaluated to ensure that no samples encountered divergent transitions, all chains exhibited convergence (rhat < 1.01) for all parameters, and no parameters exhibited low effective sample size for tail quantities (for more information on the diagnostic processes used see Supplementary material A). The datasets generated for this study can be found in the Open Science Framework (https://osf.io/qtpg5/files/osfstorage).

## Results

### Participants

No participants were excluded due to cognitive impairment (MMSE scores: *M* = 26.45; *SD* = 4.54), and participants varied greatly in both their levels of upper-extremity impairment (FM-12 scores: *M* = 8.75; *SD* = 3.02) and self-reported upper-extremity function (SIS-Hand scores: *M* = 2.57; *SD* = 1.17). Supplementary Figure 3 provides a visualization of the distribution for the MMSE, FM-12, and SIS-Hand.

### fNIRS M1-LAT

**Figure 5:**
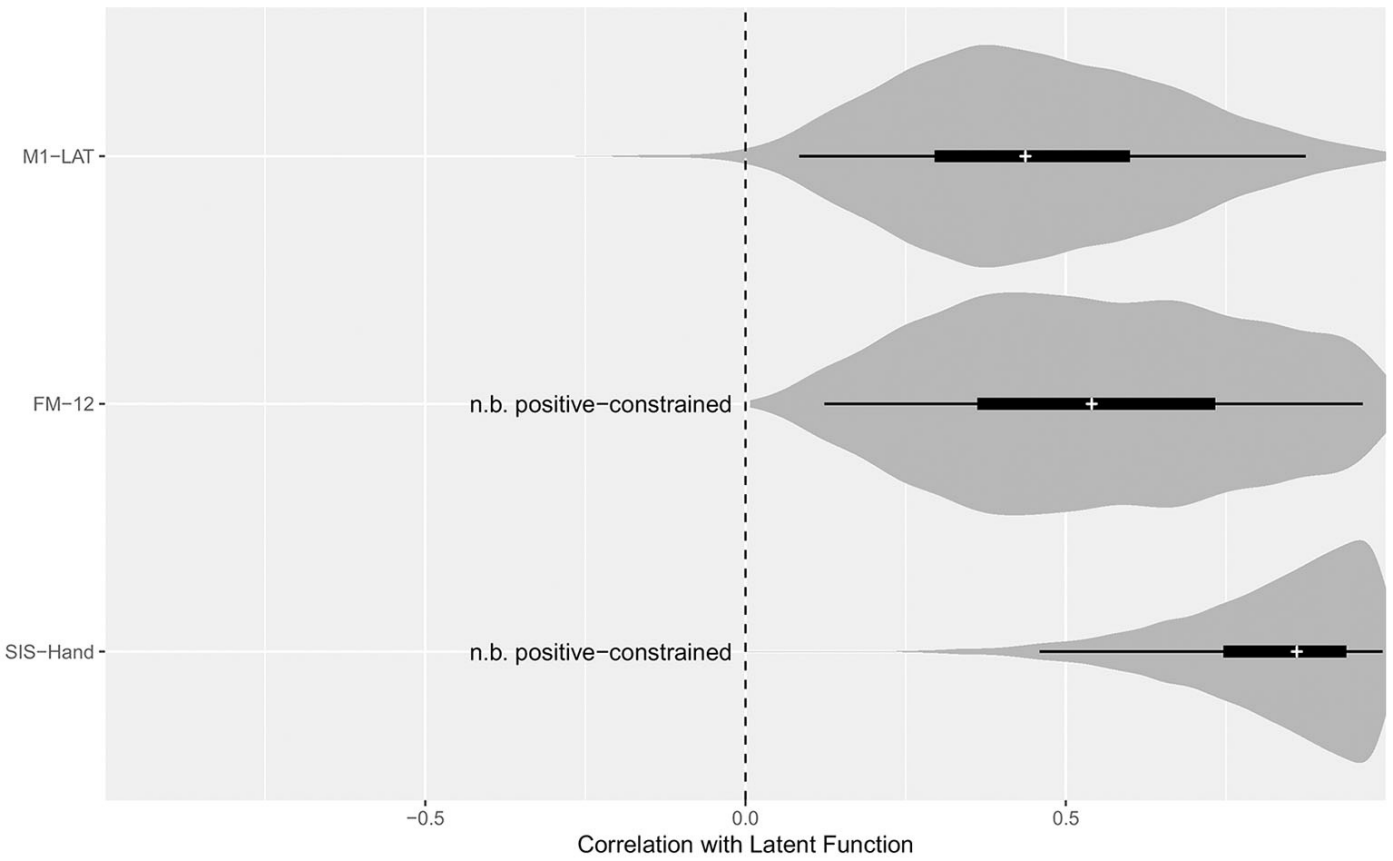
Posterior distributions for the correlation between the latent function trait and each sub-trait. Violin density functions depict the smoothed posterior density (with tail-transparency as per, Helske and Vihola, 2021), thin lines covering the CrI_95%_, thick lines covering the CrI_50%_, and medians indicated by the white cross. The SEM model constrained the potential relationship between FM-12 and latent function, as well as SIS-Hand and latent function, to be positive; while the relationship between M1-LAT and latent function was free to vary from −1 (i.e., a perfect negative correlation between M1-LAT and latent function) to +1 (i.e., a perfect positive correlation between M1-LAT and latent function).

**Figure 6:**
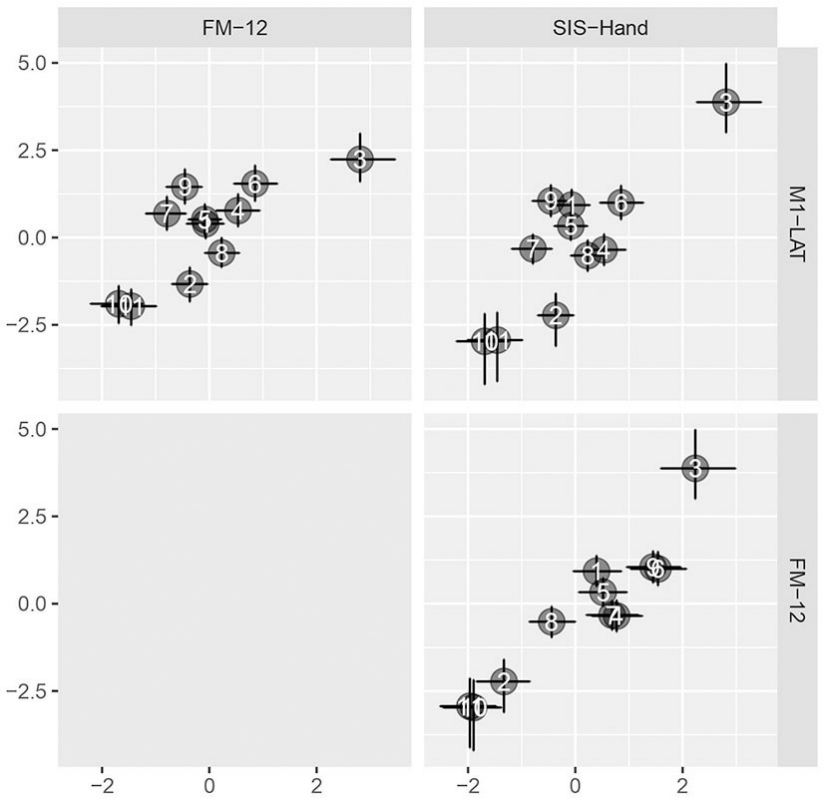
Pairwise bivariate scatter-plots of by-participant sub-traits. Horizontal and vertical error bars convey the central 50% credible interval (25%ile to 75%ile) on these constructs, crossing at the 50%ile.

As shown in Figure 5, the latent SIS sub-trait appeared to have a stronger correlation with the latent UE-IF trait than did the latent FM sub-trait, however explicit computation of the posterior for the difference did not substantially exclude zero as a credible value (CrI_95%_: −0.28 to +0.73). Furthermore, while the correlation between the latent UE-IF trait and latent M1-LAT sub-trait was not constrained to be positive, the posterior for this parameter nonetheless credibly excluded zero (CrI_95%_: +0.08 to +0.89; median: 0.44), confirming a positive correlation (see Supplementary Figure 5 for posteriors on the correlations between the sub-traits). With this model, it is possible to additionally extract posteriors associated with each sub-trait for each participant, yielding the bi-variate scatter-plot matrix shown in Figure 6.

**Figure 7:**
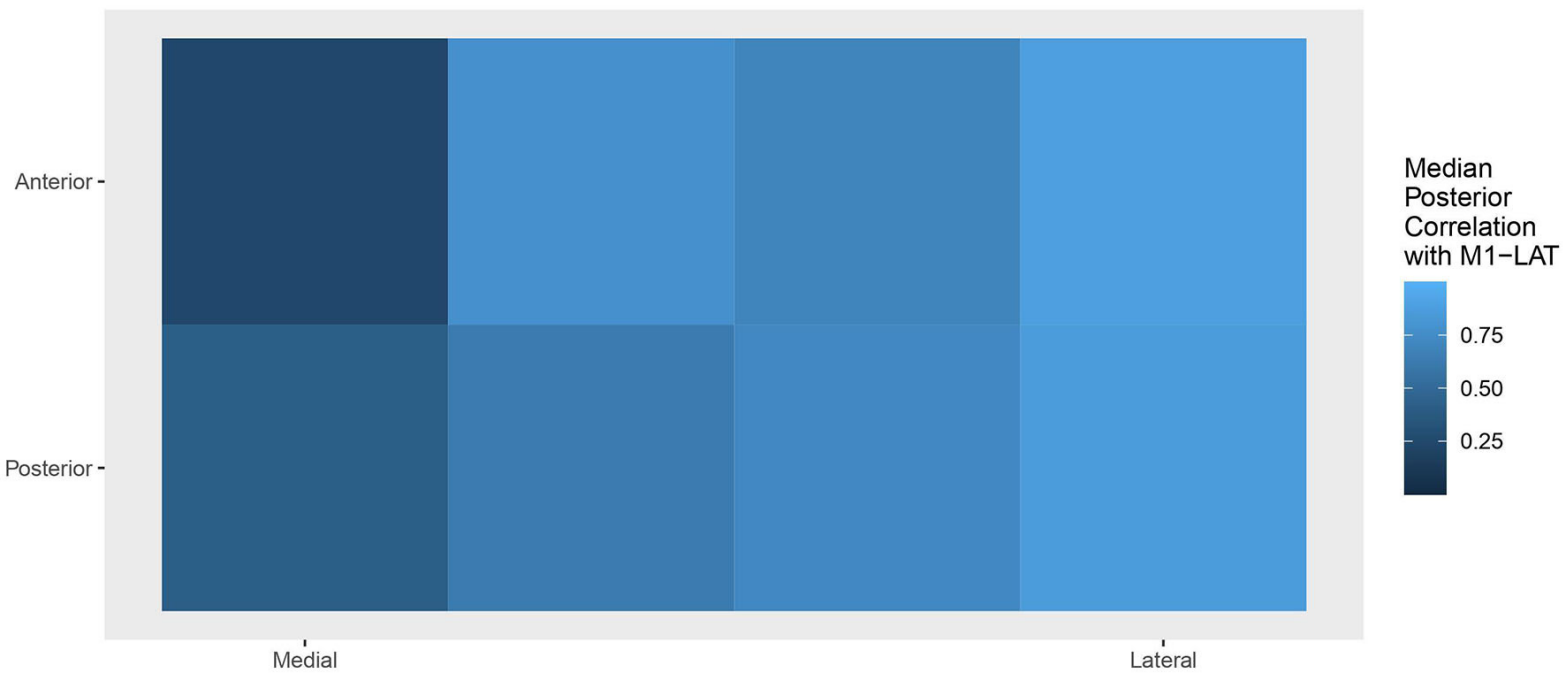
Topology of the correlations relating each location to the latent M1-LAT sub-trait, with the posterior at each location collapsed to a median. Squares on the left represent the M1-LAT values calculated between the most medial fNIRS measurement locations; whereas squares on the right represent the M1-LAT values calculated between the most lateral fNIRS measurement locations.

Figure 7 shows the topology of correlations relating each measurement location to the latent M1-LAT sub-trait, using the median of each location's posterior to determine the topological coloring, yielding a spatial pattern consistent with expectations whereby the more medial locations contain less information about the latent UE-IF trait (via the latent M1-LAT sub-trait) than the more lateral locations.

## Discussion

This study tested the ability of an ergonomic, easy-to-set-up prototype fNIRS headband to take measures of M1-LAT on stroke survivors in their homes during a simple unilateral movement; it also examined the relationship between M1-LAT (as measured during these simple unilateral upper-extremity movements) and measures of upper-extremity impairment (FM-12) and function (SIS-Hand). As expected, results showed that all participants demonstrated some event-related increases in ΔHbO across the prototype fNIRS headband's measurement grid (spanning the sensorimotor cortices). Overall, participants demonstrated either a contralateralized or bilateral response to paretic upper-extremity movement (see Supplementary Figure 2)—and moreover, that participant's level of motor cortex lateralization during unilateral movement of the paretic upper-extremity was associated with upper-extremity impairment and function. Moreover, the results demonstrated that these correlations were primarily driven by activity at fNIRS measurement locations at the lateral motor cortex (see Figure 6); this is an expected pattern given the lateral location of the hand region of the primary motor cortex (Kaas, 2004). Thus, the data from this mobile system, collected on stroke survivors in their homes, replicates several other studies that have shown cross-sectionally that a deviation from the typical pattern of M1-LAT (during simple paretic upper-extremity movement) corresponds with worse motor outcomes. Examples of such work includes an fNIRS study which used a similar hand-grasping task during which to measure M1-LAT, and characterized impairment using Brunnstrom stages (Kato, 2014), as well as studies using other modalities (e.g., fMRI, EEG, and positron emission tomography) which have shown that atypical M1-LAT correlates with worse performance on finger tapping (Calautti et al., 2007) and peg-board tasks (Zemke et al., 2003; Serrien et al., 2004), as well as worse self-reported functional abilities (as measured by the Motor Activity Log; Cunningham et al., 2015).

There are of course several important limitations to the present study that should be noted. Firstly, as mentioned above the data collected in the present study was only cross-sectional. And while there is no reason to suspect that the device used in the present study wouldn't be capable of capturing longitudinal measurements when it is able to capture these measures cross-sectionally, further work is required to determine if longitudinally captured M1-LAT data from this prototype fNIRS headband or one similar would be capable of seeing the longitudinal changes that have been reported elsewhere (Delorme et al., 2019). Another notable limitation is that while the prototype fNIRS headband was designed to enable independent use, because of the preliminary nature of this study an experimenter placed the headband on the participants [although usability data collected in another component of this study—see Chapter 4 of Friesen (2021)—suggests it may be possible to have stroke survivors use future iterations of this prototype fNIRS headband independently]; moreover, while the prototype fNIRS headband was designed to enable quick set-up, the time to set-up the device was not recorded (although this time was ~1 min)—future work on this or subsequent prototype fNIRS headbands should include this aspect in their experimental designs, enabling the continual optimization of device setup ease-of-use over time. Due to the preliminary nature of this study, only fNIRS data from a simple fist-squeezing task was presented. While existing research has shown a similar topographic pattern of increased ΔHbo across an fNIRS measurement array between movements of the hand and shoulder (Verstynen et al., 2005), future work with the prototype fNIRS headband or subsequent fNIRS prototypes ought to utilize movements involving the shoulder to replicate these results. Similarly, future work ought to include more complex upper-extremity tasks, to determine if (as has been shown via both fNIRS and fMRI) such tasks might result in a more broad pattern of motor cortex activation. Furthermore, as mentioned above, measures of M1-LAT have been shown to be relevant to lower-extremity rehabilitation as well, and fNIRS signals from the motor cortex have shown great promise as a viable data input into a lower-extremity-focused brain-computer-interface system for neurological rehabilitation (Khan et al., 2018); and while the prototype fNIRS headband used in the present study has been shown to be capable of measuring sensorimotor activity during lower-extremity activity in healthy controls (Holland, 2020), no lower-extremity tasks were collected in the present study. And finally, due to the COVID-19 pandemic, the sample size of the present study was truncated. While this undoubtedly represents a limitation, in the present context it only serves to attenuate our estimate and increase our uncertainty of the true correlation between M1-LAT and post-stroke motor impairment/function (as opposed to changing any general conclusions that might be drawn from the data).

Despite these shortcomings, the ability of this prototype fNIRS headband to measure M1-LAT in the home setting is notable. While the details of their clinical implementation has yet to be fully articulated, these measures (i.e., M1-LAT) have been shown to predict functional gains from a rehabilitation intervention (Nhan et al., 2004; Loubinoux et al., 2007; Quinlan et al., 2015; Rehme et al., 2015). Given the relevance of M1-LAT for post-stroke physical recovery, it is feasible that its ongoing collection could potentially improve clinicians ability to personalize the planned rehabilitation intervention to the needs of individual stroke survivors. For instance, given the observed association between brain-derived neurotrophic factor, post-stroke use-dependent plasticity, and motor recovery (Shiner et al., 2016; Kotlega et al., 2017; Balkaya and Cho, 2019), it is possible that ubiquitous M1-LAT measurements during post-stroke movement rehabilitation could be used to achieve a version of the “dose assignment” study Jeffers et al. performed with rats (Jeffers et al., 2018a). While it would require more clear understanding of the underlying mechanisms at play, it is intriguing to speculate about the possibility of prescribing stroke survivors a dosage of rehabilitation based on a probabilistic model of the relationship between rehabilitation dose and recovery. Such an ability would represent a pathway to scale and evolve efforts such as the Queen Square programme, which again found that even chronic stroke patients can make significant gains in motor impairment and function given a sufficiently large dose of rehabilitation. Another potential avenue of utility for ubiquitous M1-LAT measurements during post-stroke physical rehabilitation could be through their integration into the rehabilitation itself via a brain-computer-interface system, as brain-computer-interface systems using M1-LAT as feedback have been shown to increase the clinical benefits of post-stroke upper-extremity rehabilitation interventions (Mihara et al., 2013; Pichiorri et al., 2015; Ono et al., 2018). Indeed, a recent pre-print even showing that this learned M1-LAT modulation (compared to sham feedback) was not only associated with enhanced upper-extremity recovery, but with changes in structure to the ipsilesional corticospinal tract as well (Sanders et al., 2021).

Although it is worth dwelling upon the reality that realizing much of this potential will require a better understanding of the mechanisms underlying post-stroke M1-LAT presentation. The most glaring indication of this is the fact that several studies show the inverse relationship between M1-LAT and recovery—with highly atypical M1-LAT patterns (i.e., where activity is lateralized toward contralesional M1) predicting better outcomes than more bi-lateral patterns of M1-LAT (Dodd et al., 2017). This has been addressed through several mechanistic accounts of why the relationship between M1-LAT and motor recovery might be a bimodal one. Such theories span from the potential bimodal effect of interhemispheric inhibition (Bertolucci et al., 2018; Lin et al., 2020), to varying theories linking this bimodal role of M1-LAT to ipsilesional corticospinal tract integrity (Di Pino et al., 2014), to work examining the roles of alternate descending tracts originating in contralesional motor regions [such as the reticulospinal (McPherson et al., 2018; Hammerbeck et al., 2021) or rubrospinal tract (Guo et al., 2019)], to theories on the ability of neurons originating in the contralesional motor cortex to “crossover” at the level of the spinal cord (Wahl et al., 2017), to the relationship between the roles of the corticospinal with these alternate tracts (Liu et al., 2020).

However, in a classic Chicken-Egg problem, slowing down our progress on answering such questions (answering which would make the clinical utility of ubiquitous M1-LAT measurement more apparent and urgent), is the fact that to date this literature continues to rely primarily on studies using fMRI. While fMRI is surely the premier way to gain non-invasive functional measurements on cerebral activity, the use of MRI comes with significant cost (both upfront and maintenance), a large footprint (meaning stroke survivors must be brought from their site of care for these measurements to be gained), and specialized staff required to support data acquisition and analysis; these realities therefore represent a significant barrier to utilizing fMRI for any use case related to post-stroke physical rehabilitation. In addition to the substantial fMRI literature, there are also studies using headcap-based EEG (Gandolfi et al., 2018; Sebastián-Romagosa et al., 2020) and fNIRS (Kato, 2014; Delorme et al., 2019) systems to measure M1-LAT in stroke survivors. While cheaper and more user-friendly (smaller and less maintenance required) than fMRI, these systems are all laboratory-based, and require the use of a full headcap—they therefore are difficult to utilize in a variety of locations, and time-consuming to set-up. Thus, the present study is novel in that it demonstrates that it is possible to measure M1-LAT outside the laboratory with a non-headcap-based fNIRS system, potentially making it feasible for frequent, serial biomarker measurements to be taken in real-world rehabilitation settings—including the home—without overly disrupting clinician workflow or cutting into patient therapy time.

## Conclusion

The present study demonstrated that measures of M1-LAT could be taken using a mobile fNIRS system in the homes of stroke survivors, and moreover that these measurements replicate the previously described relationship between M1-LAT during paretic arm movement, and levels of impairment/function in stroke survivors. This suggests it may be possible for M1-LAT measurements to be taken on stroke survivors in more convenient and cost-effective ways than the literature to date (primarily fMRI and laboratory-based EEG and fNIRS studies) would suggest. Moreover, given previous studies that have shown that the provision of M1-LAT neurofeedback during various rehabilitation interventions can enhance the efficacy of rehabilitation (Mihara et al., 2013; Tsuchimoto et al., 2019), the present study's findings that a mobile, easy-to-use fNIRS system can take these measurements might also be built on through future development of more accessible rehabilitation brain-computer-interface systems. These findings portend the possibility of using ergonomic fNIRS devices to capture neural biomarkers of post-stroke motor recovery, and/or to be used as a part of more accessible rehabilitation brain-computer-interfaces.

## Data availability statement

The datasets presented in this study can be found in online repositories. The names of the repository/repositories and accession number(s) can be found below: Open Science Framework (https://osf.io/qtpg5/files/osfstorage).

## Ethics statement

The studies involving human participants were reviewed and approved by Veritas IRB. The patients/participants provided their written informed consent to participate in this study. Written informed consent was obtained from the individual(s) for the publication of any potentially identifiable images or data included in this article.

## Author contributions

CF: conceptualization, methodology, software, formal analysis, investigation, writing—original draft/review and editing, visualization, and project administration. ML: methodology, software, formal analysis, investigation, data curation, writing—review and editing, and visualization. TI: conceptualization, methodology, writing—review and editing, and project administration. SB: conceptualization, methodology, writing—review and editing, supervision, project administration, and funding acquisition. All authors contributed to the article and approved the submitted version.
