# Supplementary material for: Home-based portable fNIRS-derived cortical laterality correlates with impairment and function in chronic stroke
Source: Front Hum Neurosci. 2022 Dec 9;16:1023246. doi: 10.3389/fnhum.2022.1023246 (PMC9780676; doi:10.3389/fnhum.2022.1023246)
Supplement: Supplementary file 1 [file Table_1.DOCX]

Supplementary Table 1. Detailed participant characteristics

| Age | Months since last stroke | Total no. strokes | Paretic side | Dominant hand pre-stroke | MMSE (/30) |
| --- | --- | --- | --- | --- | --- |
| 67 | 37 | 1 | L | R | 30 |
| 66 | 85 | 1 | R | L | 30 |
| 81 | 16 | 1 | L | R | 21 |
| 70 | 144 | 1 | R | R | 20 |
| 72 | 120 | 1 | R | R | 18 |
| 51 | 12 | 2 | R | R | 29 |
| 53 | 43 | 1 | L | R | 27 |
| 69 | 66 | 3 | L | R | 27 |
| 60 | 42 | 4 | L | L | 29 |
| 56 | 91 | 1 | L | L | 30 |
| 39 | 62 | 1 | L | R | 30 |
| 65 | 240 | 3 | L | L | 30 |
