# Supplementary material for: Home-based portable fNIRS-derived cortical laterality correlates with impairment and function in chronic stroke
Source: Front Hum Neurosci. 2022 Dec 9;16:1023246. doi: 10.3389/fnhum.2022.1023246 (PMC9780676; doi:10.3389/fnhum.2022.1023246)
Supplement: Supplementary file 2 [file Data_Sheet_1.pdf]

Supplementary Material A

We sought Bayesian estimation of parameters manifest in this model structure, to derive the relative credibility of various values of each given both the data and whatever prior information we might have about said parameters. While the model has many parameters (e.g., the value of a given latent trait for a given participant can be considered a parameter), the structure described above constrains the vast majority. For all SEM "influence" parameters, uninformed flat priors were used. For all cutpoints, we use the "induced Dirichlet" prior.[1] Both Student-t degrees-of-freedom parameters received a prior equivalent to a parabola peaked at 15 and ranging from zero to 30 (achieved as df/30 ~ beta(2,2)). A weakly-informed/data-driven prior was achieved for the remaining fNIRS-related parameters by pre-scaling the data (subtracting a robust estimator of the mean then dividing by a robust estimator of the standard deviation) then using zero-mean/unit-variance normal priors for all mean-encoding parameters and a zero-avoiding/unit-scale (shape=2,scale=1) Weibull prior for all standard-deviation-encoding parameters.

The model and priors were expressed in Stan[2], permitting use of the cmdstan Markov Chain Monte Carlo sampler to generate posterior samples reflecting the posterior probability distributions on the model parameters given the model structure, priors and observed data. Diagnostics for all sampling runs were evaluated to ensure that no samples encountered divergent transitions, all chains exhibited convergence (rhat<1.01) for all parameters, and no parameters exhibited low effective sample size for tail quantities. We ran 6 independent chains, each for 1000 warmup iterations (during which Discontinuous Hamiltonian Monte Carlo learns the topology of the posterior distribution) followed by 1000 sampling iterations. The cmdstanr interface provides a number of diagnostic checks that can flag when the results from such

sampling are certainly untrustworthy, but our sampling passed all said checks. Specifically, no post-warmup divergent transitions were encountered, energy Bayesian fraction of missing information was above .3 for all transitions, Rhat values were below 1.01 for all parameters, and effective sample sizes for both bulk and tail quantities of all parameters were above 1000.

Supplemental Figure 1. Overview of pre-processing procedures

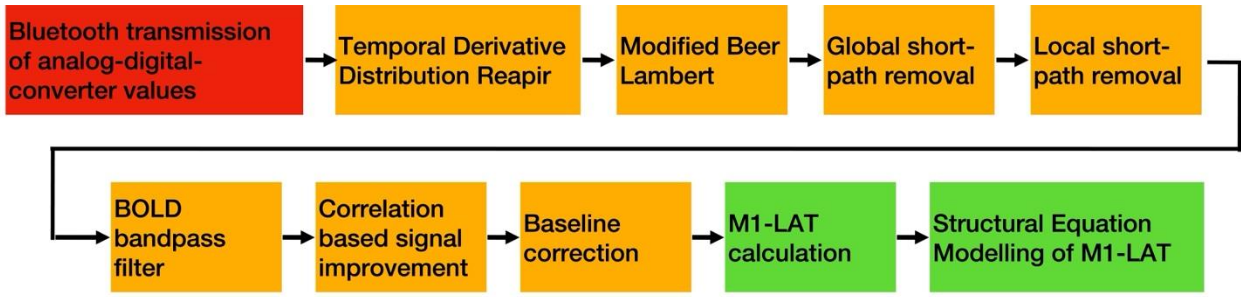

Supplemental Figure 1. The sequence of processing steps to go from raw fNIRS data transmission to the processed data inputs of M1-LAT that were used in a structural equation model (see section 2.8 and Figure 3 for details on this process). Red represents the initial transmission step, orange represents pre-processing steps, while green represents post-processing steps.

Supplemental Figure 2

Given the hypothesis that the degree of M1-LAT would correlate with stroke survivors' level of upper-extremity impairment and function, we expected the topographical distributions of event-related ΔHbO patterns to vary between individuals (with more contralateralized patterns for stroke survivors with lower levels of impairment)—though with strong priors that we would

see a task-evoked increase in at least a sub-segment of measurement locations, in at least one hemisphere.

To visualize a representation related to the average timeseries for each measurement location and each participant, the ΔHbO time series data were collapsed across trials (within each measurement location) to a mean timeseries using a generalized additive model (GAM). A GAM was then fit by generalized cross-validation, resulting in a single timeseries for each participant for each measurement location, and the 95% confidence ribbons of a mean time course at each location was preserved.

In looking at the topographical distribution of mean ΔHbO values as determined by the GAM model, indeed, we generally see a mixture of typically contralateralized increases in ΔHbO during the task period (e.g., participants 5, 7, and 11), as well participants with a more bilateral pattern of activation (e.g., participants 3, 8, and 12). As a result, in examining the posterior samples for the group mean slope obtained for the task window, as with the GAM-generated mean time-series, we see a generally bilateral increase in ΔHbO (Figure 4, main paper), with a slight contralateralization.

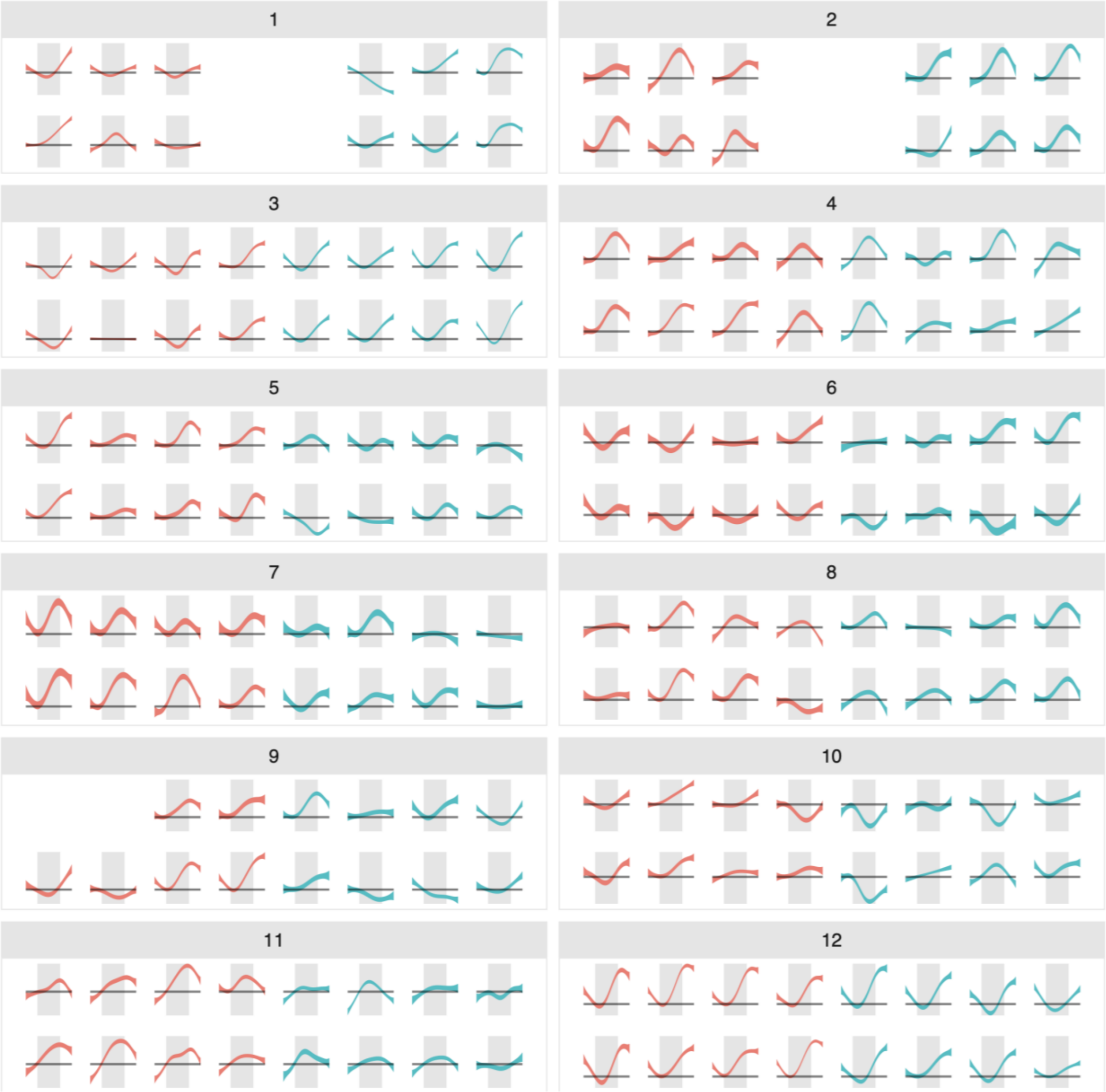

Supplemental Figure 2. Estimated 95% confidence ribbons for ΔHbO timeseries for all participants. Time series windows with red confidence ribbons are contralateral to the hand being used in the task (their stroke or ipsilesional hemisphere); time series windows with green confidence ribbons are ipsilateral to the hand being used in the task (their healthy or contralesional hemisphere). The portion of the time series shaded in grey represents the 10s block of ~1Hz fist squeezing or attempted fist squeezing. Visualized ribbons were obtained from

a GAM fit separately to each participant and measurement, and specifically represent ±2 standard error of the mean, resulting in a 95% confidence interval.

Supplementary Figure 3

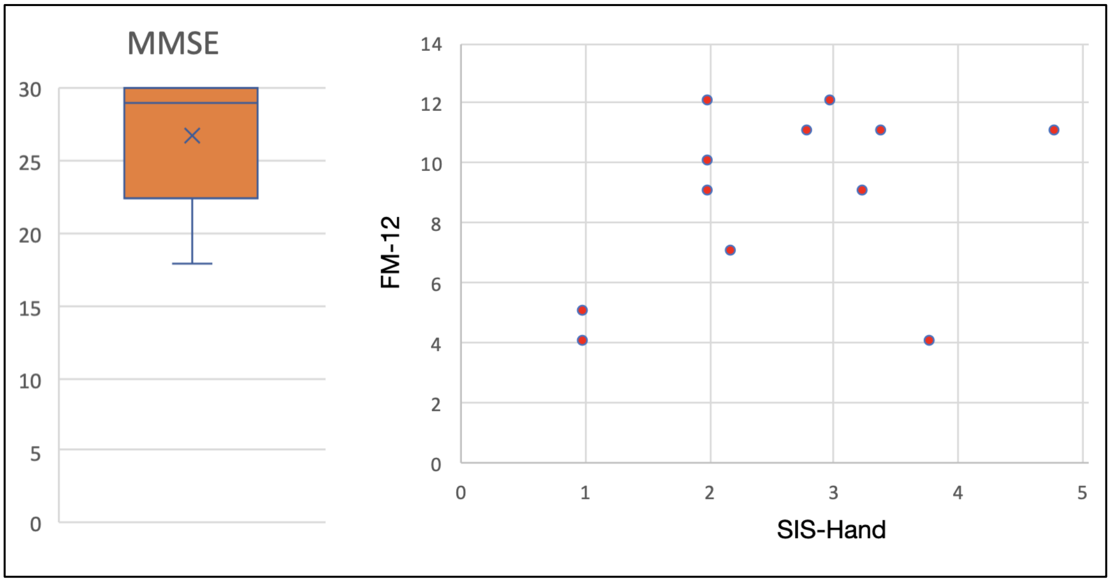

Supplemental Figure 3. Left – participants MMSE scores; the blue X within box represents mean, whereas the blue line represents the median. Right – participant scores on the SIS-Hand and FM-12.

Supplemental Figures 4.1 – 4.5

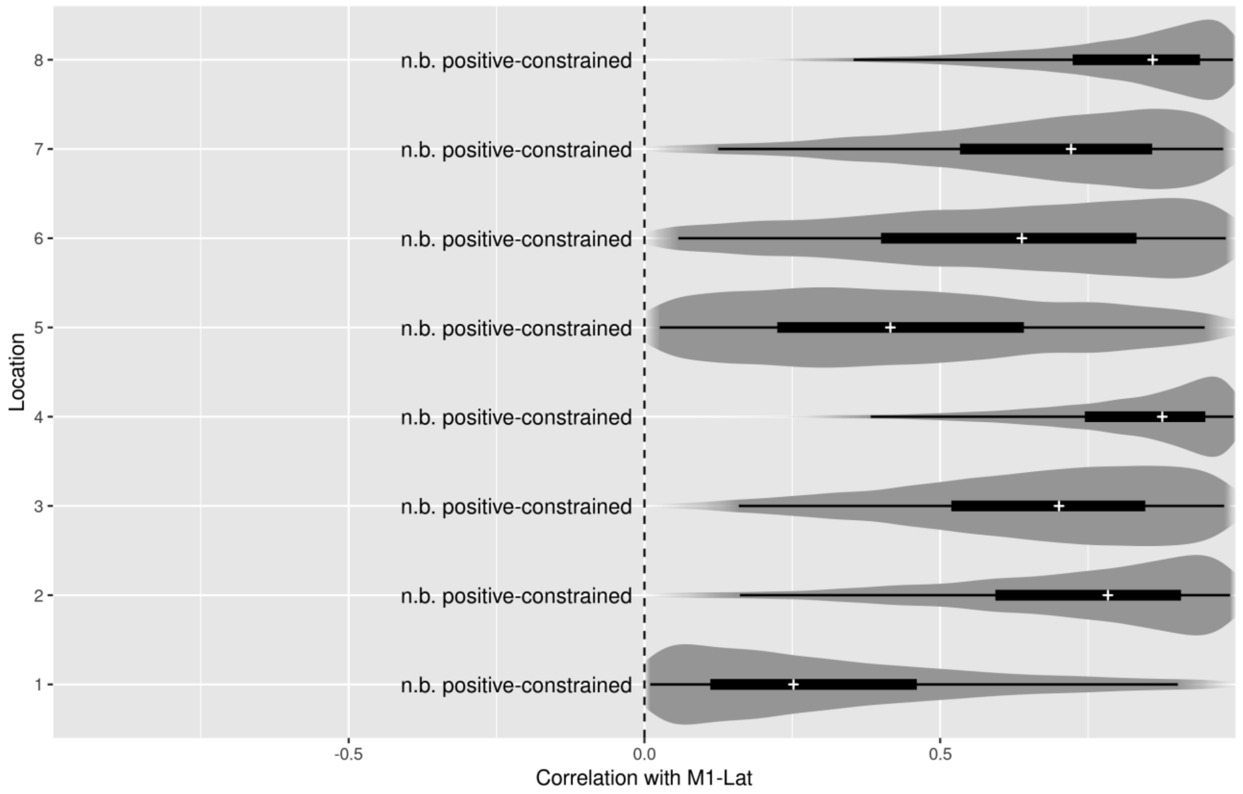

Supplemental Figure 4.1 Posterior for the correlations between the latent M1-LAT sub-trait and each measurement location.

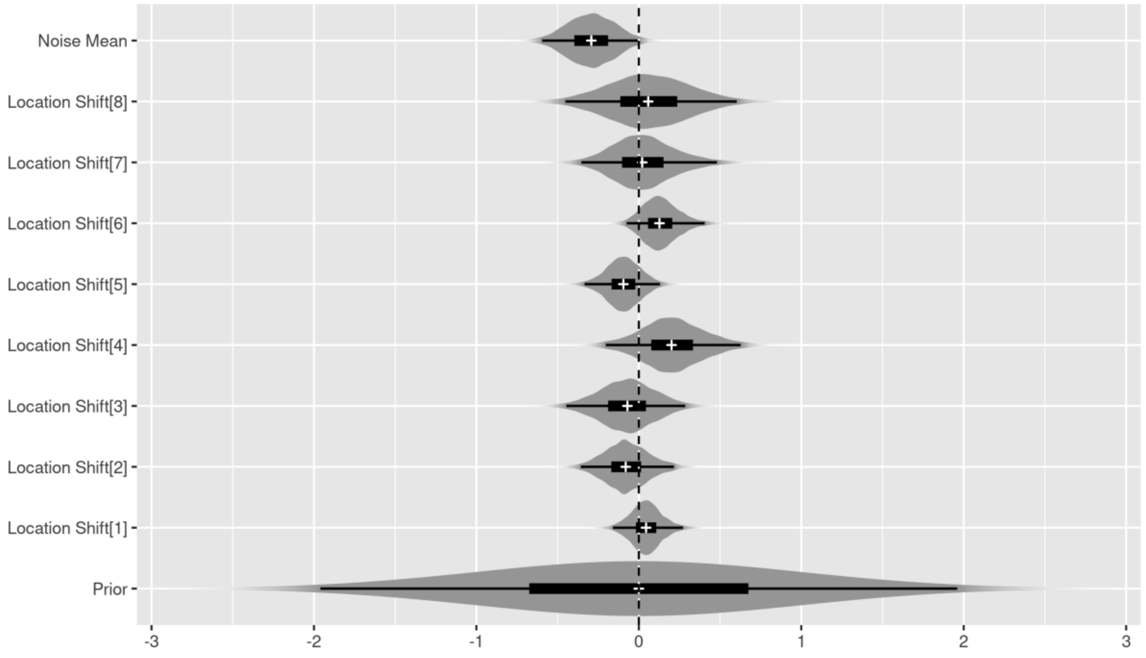

Supplemental Figure 4.2. Prior and posterior for all central-tendency parameters.

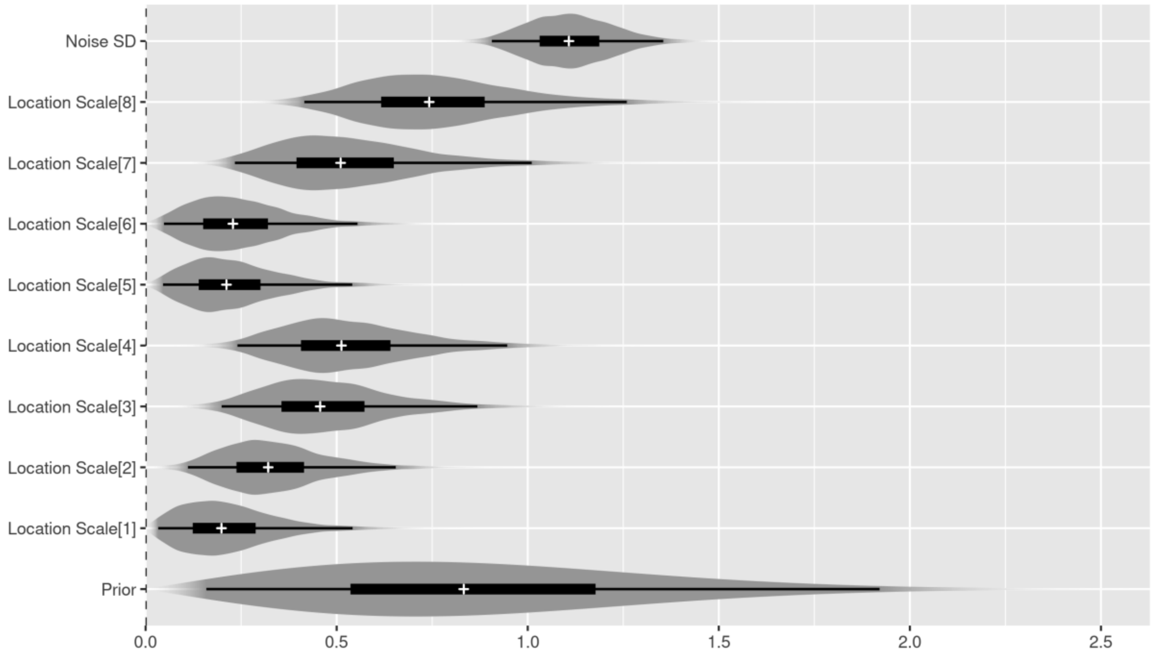

Supplemental Figure 4.3. Prior and posterior for all variability parameters.

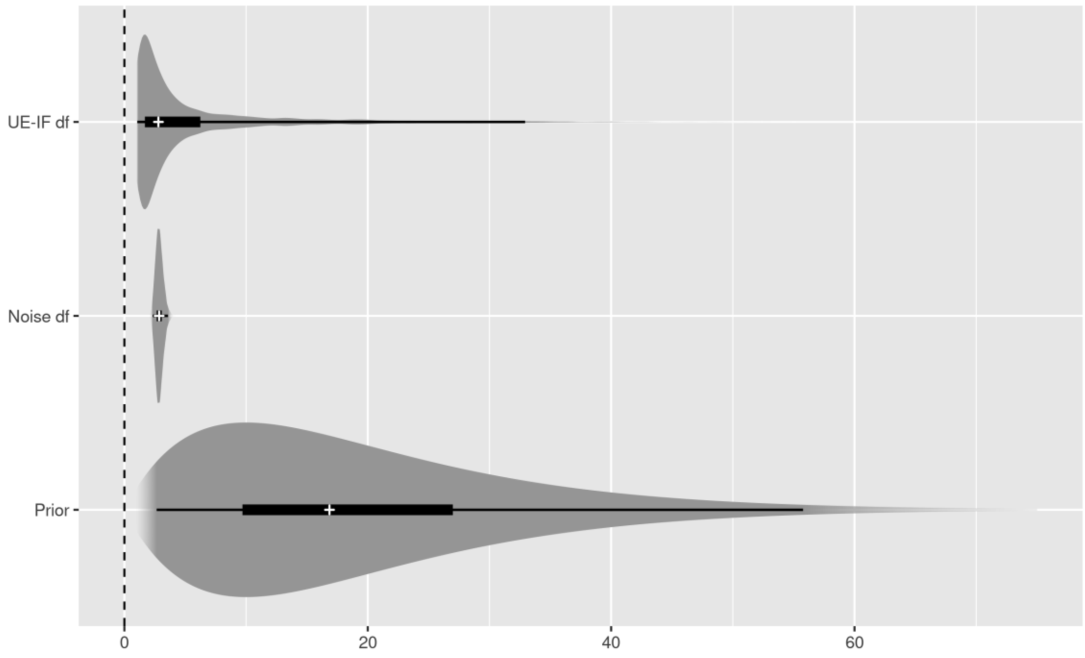

Supplemental Figure 4.4. Prior and posterior for all Student-*t* degrees-of-freedom parameters.

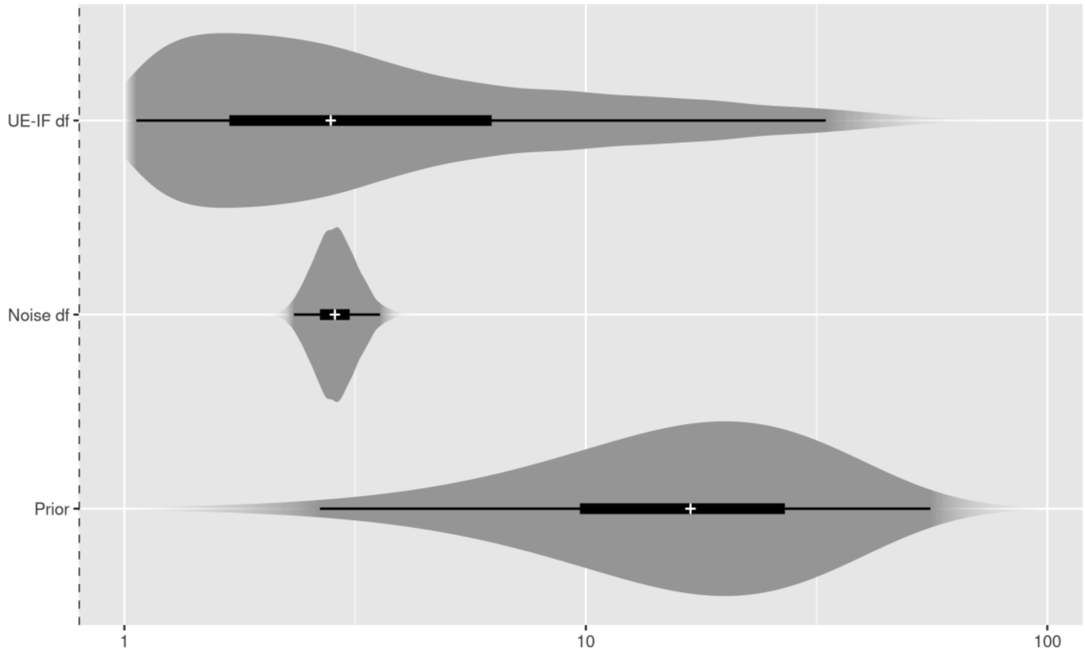

Supplemental Figure 4.5. Log scale version of the prior and posterior for all Student-*t* degrees-of-freedom parameters.

Supplementary Figure 5

With an SEM structured as we have done here (with edges parameterized by correlations), it is possible to compute the expectation for the correlation between any node by obtaining the product of edges tracing the path between them. Thus, while Figure 4 (main paper) shows the correlations between each sub-trait and the latent Function trait, we can also derive correlation among the sub-traits themselves, yielding the posteriors shown in Supplementary Figure 5 below

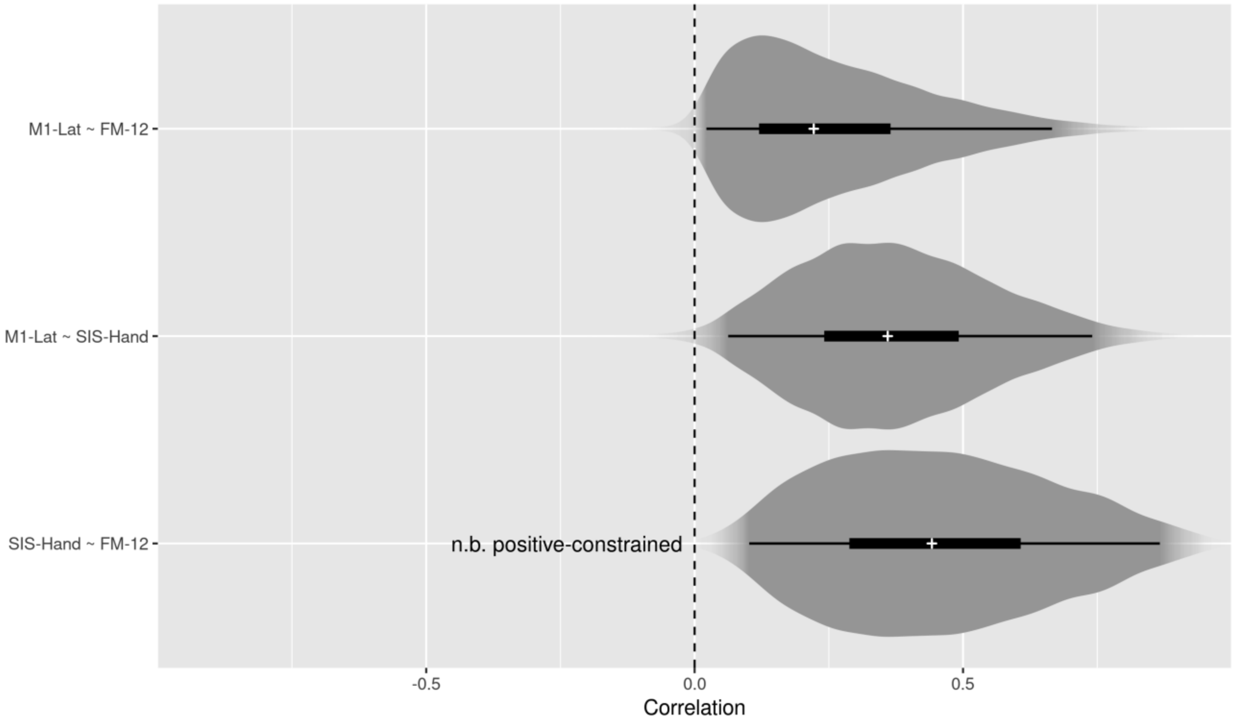

Supplementary Figure 5.

<u>References</u>

1. Betancourt, M. Case Studies: Ordinal Regression.
2. Gelman, A., Lee, D. & Guo, J. Stan: A Probabilistic Programming Language for Bayesian Inference and Optimization. *J. Educ. Behav. Stat.* 40, 530–543 (2015).
